# The dentin organic matrix – limitations of restorative dentistry hidden on the nanometer scale

**DOI:** 10.1016/j.actbio.2012.02.022

**Published:** 2012-07

**Authors:** Luiz E. Bertassoni, Joseph P.R. Orgel, Olga Antipova, Michael V. Swain

**Affiliations:** a Biomaterials Science Research Unit, Faculty of Dentistry, University of Sydney, United Dental Hospital, Surry Hills, Sydney, NSW 2010, Australia; b Biophysics Collaborative Access Team, Center for Molecular Study of Condensed Soft Matter, Pritzker Institute of Biomedical Science and Engineering, Center for Synchrotron Radiation Research and Instrumentation (CSRRI), Department of Biological, Chemical and Physical Sciences, Illinois Institute of Technology, Chicago, IL 60616-3793, USA

**Keywords:** Dentin, Collagen, Bonding, Adhesion, Polymers

## Abstract

The prevention and treatment of dental caries are major challenges occurring in dentistry. The foundations for modern management of this dental disease, estimated to affect 90% of adults in Western countries, rest upon the dependence of ultrafine interactions between synthetic polymeric biomaterials and nanostructured supramolecular assemblies that compose the tooth organic substrate. Research has shown, however, that this interaction imposes less than desirable long-term prospects for current resin-based dental restorations. Here we review progress in the identification of the nanostructural organization of the organic matrix of dentin, the largest component of the tooth structure, and highlight aspects relevant to understating the interaction of restorative biomaterials with the dentin substrate. We offer novel insights into the influence of the hierarchically assembled supramolecular structure of dentin collagen fibrils and their structural dependence on water molecules. Secondly, we review recent evidence for the participation of proteoglycans in composing the dentin organic network. Finally, we discuss the relation of these complexly assembled nanostructures with the protease degradative processes driving the low durability of current resin-based dental restorations. We argue in favour of the structural limitations that these complexly organized and inherently hydrated organic structures may impose on the clinical prospects of current hydrophobic and hydrolyzable dental polymers that establish ultrafine contact with the tooth substrate.

## 1 Introduction

The prevention and treatment of tooth decay are major challenges in dentistry. It is estimated that 90% of adults in Western countries suffer from this dental disease [1], and in the USA alone the annual expenditure associated with dental services surpasses $100 billion dollars [2]. Although preventive and restorative dentistry has undergone significant advances in recent years [3–7], a primary reason driving this economic burden is the placement and replacement of tooth fillings due to the low durability of current polymeric restorative dental materials, particularly when damaged dentin is involved [8].

Dentin is a hierarchically organized nanostructured biological composite that combines highly complex protein assemblies to form a strong and durable mineral-rich biological material. The primary objective of restorative dentistry is to repair and replace damaged tooth structures by the systematic application of synthetic materials aiming at the re-establishment of tooth aesthetics and function. Current restorative procedures generally depend on the formation of an adhesive bond between polymeric dental materials and the tooth substrate [5]. Therefore, current tooth coloured restorations rely substantially on the ultrafine interaction of synthetic polymers with the highly complex supramolecular assemblies that compose the tooth organic matrix. This is generally described as restorative adhesive dentistry.

Improvements in our ability to understand the physical processes on the nanometer scale in recent years has raised critical questions regarding the long-term effectiveness of current restorative materials [9]. The dentin organic matrix has been largely explored and novel findings point to a highly complex and somewhat unfavourable interaction between dental materials and its organic constituents from a nanostructural perspective [5,10–12]. In this review we discuss recent evidence on the structural organization of the dentin organic matrix on the sub-micrometer scale. We support the perspective that the paradigms that currently dictate restorative dentistry are founded on structural, molecular and biological phenomena that impose critical limitations on the long-term prospects of polymeric dental restorations. Additionally, we argue that the dentin matrix is comprised of highly orchestrated protein assemblies that present extremely complex organizational features at the nanoscale and at the molecular level which show intricate interactions with polymeric restorative materials, particularly currently used dental adhesives.

## 2 The dentin substrate and the adhesion phenomenon

Dentin is the tissue underlying the dental enamel that constitutes the bulk of the tooth. It has a specifically oriented micro-morphology composed of tubules (∼1–2 μm diameter) [12] surrounded by a hypermineralized layer (∼1 μm), called peritubular dentin, and a softer intertubular matrix, where the organic material is concentrated [13]. The intertubular matrix is mainly composed of type I collagen fibrils with associated non-collagenous proteins and proteoglycans, forming a three-dimensional organic network reinforced by apatite mineral crystallites [12]. The apatite in dentin, in contrast to enamel, is partitioned according to its location with respect to the collagen fibrils into extrafibrillar mineral, located in the spaces separating the fibrils [14–16], and intrafibrillar mineral, mainly in the gaps regions within the fibrils extending between collagen molecules [17,18].

The repair of tooth structures with polymeric adhesive materials generally involves three distinct processes [5], namely acid etching, priming, and bonding, although several self-adhesive resins also exist. A wide range of organic and inorganic acids have been investigated as etchants, and strong phosphoric acid gels (pH ∼1.0) have been shown to produce the most reliable etching patterns [19,20]. Primers, on the other hand, are an assembly of hydrophilic monomers and volatile solvents, usually acetone, ethanol–water or primarily water, which are used to displace the fluids from the dentin matrix and carry the monomers into the demineralized collagenous network [5]. The microporosity created by the acid etching procedure is then infiltrated with monomeric dental adhesives that are intended to surround and embed the remnant protein-rich substrate, thus providing adhesion to the tooth structure via micromechanical retention [5]. In dentin, infiltration of the adhesive resin into the collagen network is a process termed hybridization [21–23]. The result of this diffusion process has been called the resin inter-diffusion zone or simply the “hybrid layer”. The adhesive resins are generally hydrophobic dimethacrylate oligomers diluted with low molecular weight monomers which are compatible with monomers in the primer and the resin material that is subsequently used for reconstruction of the tooth morphology.

In summary, the infiltration of synthetic monomers within the organic scaffold in dentin forms the basis for a large variety of restorative procedures currently performed in dentistry [8]. For instance, the majority of cavity restorations performed nowadays use toothcoloured composite resins that are bonded to the tooth substrate with an adhesive material via hybridization. Similarly, polymer-based cements rely primarily on the interaction of dimethacrylate monomers with the dentin substrate, i.e. resin modified glass ionomers, liners, cements, etc. It is generally accepted that the final goal of these adhesive procedures is the complete infiltration and encapsulation of the demineralized collagen fibrils (and other remnant organic structures in the tooth structure) by the monomeric resin [5,10,21–24]. Unfortunately, this paradigm underestimates the considerable complexity of the supramolecular structures that form the dentin matrix on the nanometer scale. For instance, the so-called nanoleakage phenomenon, which has been described as the main degradation mechanism of tooth–biomaterial interfaces [25], has been shown to result from the poor ability of monomers to impregnate the demineralized dentin organic matrix. This has been demonstrated to facilitate water sorption and hydrolytic degradation [26], even in the absence of a visible marginal gap at the tooth–biomaterial interface [27].

The following topics will address the complexity of the organic structures in the dentin matrix on decreasing sub-micrometer scales. More specifically, we will discuss critical boundaries for the effectiveness of the interaction of polymeric dental materials with dentin in the light of their complex nanostructural interactions.

## 3 The dentin organic matrix: supramolecular assemblies with complex organizational features on sub-micrometer scales

It is generally accepted that 90 wt.% of the organic phase in dentin is almost exclusively composed of collagen type I [28,29], although other types of collagen have also been identified [28]. The remainder of the dentin organic matrix is composed of non-collagenous structures, of which proteoglycans represent the entity with the better known structural and mechanical relevance; other dentine matrix proteins, such as phosphoproteins and γ-carboxyglutamate-containing proteins, are believed to be more involved in mineral–matrix binding events [30]. Given their structural relevance and relative higher abundance in the matrix the following discussion will primarily address the structural features of collagen type I, proteoglycans and the supramolecular assemblies formed by their mutual association in the matrix.

### 3.1 Collagen type I

Collagen type I represents an intricate and highly orchestrated supramolecular assembly of substructural units hierarchically organized on the sub-micrometer scale forming larger fibrils. The complexity of the molecular interactions in collagen type I increases gradually with decreasing length scales, therefore, in this review we will address the structural features of collagen from a micrometer down to a molecular perspective.

The overall topography of a collagen fibril is as important as its internal organization. A variety of investigations utilizing a wide range of microscopy and other techniques have been devoted to understanding the morphological features of dentin collagen, including small angle X-ray scattering [31], scanning electron microscopy (SEM) [32], field emission SEM [33], immunocytochemical SEM [34,35], transmission electron microscopy (TEM) [36] and atomic force microscopy (AFM) [37–40]. Nevertheless, the majority of the studies to date have primarily focused on the structural features of collagen at the fibrillar level. Hence, the more complex and equally relevant nanoscale substructural units that extend beyond the collagen D-periodical topographical features have largely been underestimated in dentistry thus far. From a mechanistic perspective the criticality of this knowledge gap lies in the assertion that the final goal of general adhesive procedures is the complete infiltration and encapsulation of the collagen fibrils [5,10,24,41]. Thus an improved understanding of the organizational features of the supramolecular assemblies that constitute a collagen fibril and its interactions with polymeric dental materials is imperative. To the best of our knowledge, however, there have been no attempts to collectively address these aspects in the current literature.

#### 3.1.1 The fibrillar level – topographical features and limitations for an improved interaction with dental monomers

An important observation that needs to be carefully clarified prior to further discussion on the hierarchical organization of dentin collagen is that the terms “collagen fibril” and “collagen fiber” have been used interchangeably in the literature. We highlight that collagen “fibers” are limited to larger (∼10 μm) collagen aggregates, such as the ones found in bone, which are essentially comprised of thinner (∼100–200 nm) “fibrils”. We contend, therefore, that the accurate nomenclature for the D-periodical organic structures found in dentin should be “collagen fibrils”, since etymologically the term “fiber” refers to larger fibrillar entities, whilst the term “fibrils” originates from thinner filaments or “fibrillas”.

Several electron microscopy studies have determined the collagen fibril diameter to be around 100 nm [20,42,43], however, values as low as 20–60 nm have also been reported in the literature [32,44]. A noteworthy study by Habelitz et al. [37] used AFM to diligently analyse a total of 395 and 180 diameters of individual fibrils in the hydrated and dehydrated states, respectively. This investigation identified three distinguishable sizes, 83, 91, and 100 nm, for the hydrated fibrils, while dehydration caused a broad distribution between 80 and 100 nm [37].

**Fig. 1:**
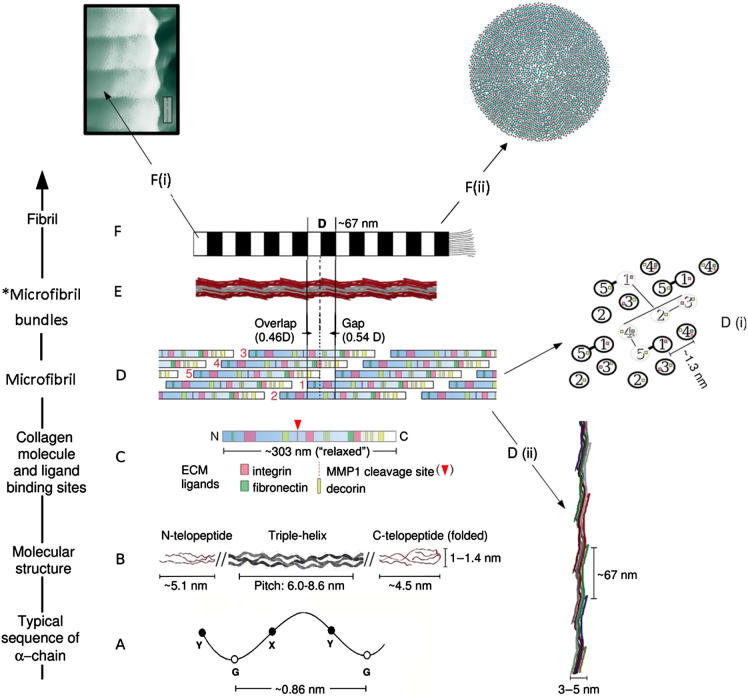
Increasing complexity of the organizational hierarchy of collagen type I (modified from Orgel et al. [62]). (A) Collagen molecules are composed of three α polypeptide chains; one chain is shown. The repeating (X–Y–Gly)*_n_* pattern in which the X and Y positions are frequently occupied by proline and 4-hydroxyproline residues is represented by X, Y and G. (B) A not to scale (shortened) illustration of the collagen monomeric molecular structure depicting the non-helical N- and C-telopeptides bordering the long, central, helical domain. (C) Molecules are approximately 303 nm long (the relaxed length is a straight line measurement from end to end). Four collagen–ligand binding sites are indicated. (D) Simplified collagen molecular lateral packing: each molecule is staggered from its neighbour by a multiple of ∼67 nm. The gap region is where there are four collagen molecular segments and the overlap region where there are five. (D-ii) Schematic two-dimensional representation of the lateral molecular packing (D) and microfibril topology (light grey) illustrating the quasi-hexagonal arrangement. The intermolecular separation is slightly more or slightly less than 1.3 nm inside the hydrated fibrils, yielding a molecular packing that is quasi-hexagonal. (D-ii) Each collagen molecule in the microfibril is coloured so that it is obvious that each D-period contains molecular segments from five different molecules. (E) Three interdigitated microfibrils where each red and grey microfibril bundle represents a single microfibril, as shown in D(ii), forming an intermolecular association that would resemble thinner microfibrillar bundles (provided that these are not a random disaggregation event) (F) The type I collagen fibril exhibits a characteristic periodic banded pattern originating from the presence of a gap (black) and an overlap region (white) in the collagen axial packing (D). (F-i) AFM micrograph of a collagen fibril. (F-ii) Lateral view of the molecular packing within a single fibril, where each circle represents the estimated position of each collagen molecule in cross-section (adapted from Hulmes et al. [71]).

A feature that distinguishes collagen from other fibrillar macromolecules is that they are most easily recognized by their axial 67 nm periodicity [45], which can be seen by AFM [37,40] and electron microscopy [10,35,36] and can also be inferred from X-ray diffraction (XRD) data [46–52]. The 67 nm periodicity (corresponding to one D-period) stems from the staggered arrangement of collagen molecules in a given fibril, where the staggered spaces between the ends of successive collagen molecules yield the so-called gap zones and the areas where multiple molecules are superimposed represent the overlap zone (Fig. 1).

AFM studies have determined that the height distance between the gap and the overlap zones increases with demineralization, which has been shown to occur due to more rapid dissolution of mineral in the extrafibrillar than in the intrafibrillar compartment [17]. Accordingly, the height distance between the gap and overlap zones in fully demineralized collagen was shown to range from 4 [37] to 6 nm [17], although in theory the collagen molecular packing difference should account for only about a 2.5 nm height distance, considering that the C-teleopepide is thicker than a collagen molecule, and the orientation of the molecules transitioning from the overlap to gap phase changes slightly. Moreover, it has been demonstrated that after demineralization and subsequent treatment with NaOCl the gap and overlap zones of adjacent fibrils appear to interlock and form tight junctions, even when the tissue is fully immersed in water [37].

**Fig. 2:**
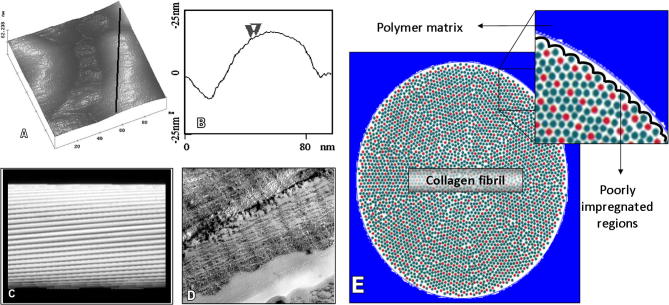
Topological features of collagen type I. (A) Tapping mode AFM image obtained in liquid of an individual gap zone of a dentin collagen fibril and the adjacent overlap zones. (B) Section analysis across the diameter of a fibril overlap zone reveals “bumps” at about 4 nm distance that have been associated with collagen microfibrils (A and B adapted from Habelitz et al. [37]). (C) Molecular model of the microfibrillar arrangement of collagen type I. (D) The same arrangement is shown in a freezefracture micrograph of hydrated, unfixed collagen type I from rat tail tendon with a horizontal field of view of 500 nm (C and D adapted from Ottani et al. [142]). (E) Schematic representation of the radial packing of collagen molecules (adapted from Hulmes et al. [71]) showing a fibril surrounded by the polymeric matrix illustrating the difficult hermetic enveloping of collagen by viscous monomers due to the presence of ∼4 nm surface “bumps”.

Surface features suggestive of the presence of collagen substructural units, commonly described as either microfibrils or subfibrils, have also been identified in dentin. We contend that microfibrils may be a more appropriate nomenclature for these thinner structures, as the features identified as subfibrils by microscopy generally refer to microfibrils earlier identified by XRD studies, which offer a much broader and earlier range of reports. Habelitz et al. [37] used AFM to report features of about 4 nm in width on the surface of dentin collagen, consistent with the longitudinal microfibrils found in collagen type I of a fully hydrated tendon [53] (Fig. 2A). These surface features are difficult to quantify but have been reported to wind axially along the fibrils at a shallow angle close to 5° (Fig. 2) [53,54].

From a mechanistic perspective one can infer that the topographical features that become evident after the removal of mineral from the collagen surface, namely (1) the 4–6 nm height distance between the gap and overlap zones, (2) the surface microfibrils about 4 nm in width and (3) the tight junctions of interlocked gap and overlap zones in adjacent demineralized fibrils, may hamper the putative complete infiltration and encapsulation of collagen fibrils in dental adhesive procedures. A complete and precise enveloping of the nanoscale irregularities on the collagen fibril surface by the passive mechanism of penetration and adsorption of the viscous monomers that compose adhesive systems and other polymeric dental materials, such as dental cements and monomer-containing glass ionomers, is unlikely to occur. The ability of polymeric resins to fully occupy the crevices present on the collagen fibril surface, such as the 4–6 nm deep gap zones and ∼4 nm wide microfibrils, depends heavily on the low viscosity of the monomeric material and the pristine action of the primer molecules in altering the surface energy of the fibril. Nevertheless, even the least viscous resin with the most effective primer will encounter difficulties in hermetically surrounding these nanometer scale features, as schematically depicted in Fig. 2.

The voids formed by the putative inappropriate enveloping phenomenon may be on the sub-nanometer scale in the early stages, and thus are hardly detectable with current microscopy techniques. Nevertheless, they may facilitate the diffusion of small water molecules, which have a diameter of about 100 pm, from the highly hydrated dentin substrate underneath restorations. It has been demonstrated that a high level of porosity and micro-voids may facilitate fluid transport into and out of the polymer by serving as sites for water molecules to be sequestrated, thus leading to enhanced solvent uptake and elution [55]. Thus these poorly enveloped regions will almost certainly represent sites where hydrolytic degradation may initiate. A large amount of evidence of micro- and nano-leakage in restored dentin supports this assertion [5,56]. The role of water in the molecular structure of collagen, which also supports this perspective, will be discussed in a separate section.

#### 3.1.2 The subfibrillar level – evidence of substructural units in the form of thinner microfibrillar bundles

As mentioned above, the description of collagen subfibrils and microfibrils has been the subject of much confusion and limited debate in the literature. Microfibrils are discontinuous 5-mer repeats of collagen molecules with diameters of around 4–5 nm, as widely documented by XRD studies [48,50,57–61]. These same structures have been commonly described as subfibrils when visualized by microscopy.

Despite the significant level of terminological confusion mentioned above there is strong evidence that collagen type I retains sub-structural fibrillar units measuring roughly 10–25 nm in diameter that may represent another structural component of collagen type I. A recent review [62] has provided a broad classification for any collagen fibril that maintains its aggregate structure by bundling smaller units together to be a “fibril bundle”. Thus, in accordance with the classification described in this review [62], the respective 10–25 nm diameter substructural units could be classified as “microfibrillar bundles”, which are thinner than the D-periodical fibrils and are constituted of aggregates of single microfibrils. Thus, in the hierarchical organization of collagen the size of these substructural units fall below the D-periodical ∼100 nm fibrils and one level above the ∼5 nm thinner microfibrils (Fig. 1). It is important to highlight, however, that these thinner substructures may not be another hierarchical level *per* se. The thinner substructures described above may rather represent transient aggregates of microfibrils that disaggregate due to environmental changes or other external events, such as thermal fluctuations within the fibril or the removal of surface bound proteoglycans.

Scott pioneered the debate concerning thinner substructural units in collagen type I [63]. In an early study he presented fibrillar entities of about 25 nm in diameter that were revealed as smaller units of larger fibrillar assemblies from cartilage. A more recent study by Yamamoto et al. [64] revealed the substructural arrangement of corneal collagen using SEM and AFM after acetic acid treatments. Structures with a diameter of about 10 nm were clearly resolved and thinner fibrillar collagen disaggregates resembling an “untwisted rope” structure were also reported. Nevertheless, comparisons with similar 10–25 nm collagen substructural units from other tissues were not established. A noteworthy study by Raspanti et al. [65] investigated the self-assembly of acid-soluble type I collagen in the presence and absence of the proteoglycan decorin, which is the most highly expressed proteoglycan in dentin. This study revealed individual substructural units and intermediate aggregates of slender filaments winding to form larger D-periodical fibrils. Accordingly, in all cases the fibrils were clearly a result of the lateral aggregation of elongated subunits of a uniform diameter of approximately 12–15 nm [65].

**Fig. 3:**
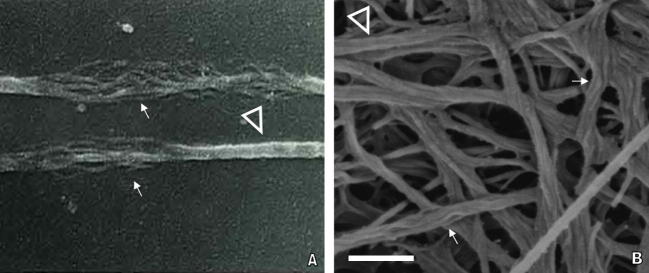
Collagen fibril disaggregation unravelling thinner collagen internal substructural units. (A) SEM image of corneal collagen fibrils treated with acetic acid and dissociated into thinner (∼10 nm) fibrillar entities (no scale bar reported, ×79,000) (adapted from Yamamoto et al. [64]). (B) Demineralized dentin collagen fibrils treated with trypsin yielding an untwistedrope like appearance and unravelling ∼20 nm substructural fibrillar disaggregates (200 nm scale bar, ×160,000).

More specifically to dentin, we have recently obtained evidence of demineralized collagen following treatment with trypsin, which clearly unravelled consistent 20 nm thin fibrillar units originating from larger D-periodical fibrils (Fig. 3). The resemblance between the “untwisted rope” appearance reported by Yamamoto et al. [64], as well as the structures described by Scott [63] and Raspanti et al. [65] with the thinner fibrillar disaggregates we found is striking (Fig. 3). Scott hypothesized that the disaggregation of larger fibrils into thinner fibrillar entities demonstrates that there are characteristic aggregates of collagen molecules which are more stable than thicker “parent” fibrils [63]. Accordingly, collagen microfibrils (∼5 nm diameter) may assemble into concentric bundles, thus forming substructural units 10–25 nm in diameter, perhaps in a transient state, and the interactions within the assembled bundles would be stronger than those between them, since the microfibrillar bundles themselves did not disaggregate under conditions sufficient to cause unravelling of the larger D-periodical fibrils [63].

An interesting aspect of this discussion is that collagen fibrils of very young connective tissues, such as tendons, are much thinner than those of mature tissues. They have fairly narrow size distributions, with diameters averaging about 20 nm, and tend to thicken via lateral aggregation of fibrillar units over time [66]. Thus the trypsin digestion mentioned above unravelled 20 nm structures that are consistent with the lateral step-wise growth of developing collagen [66]. Moreover, a noteworthy observation is that reports of thinner collagen disaggregates in the literature generally present fibrillar features with diameters that are multiples of 4 or 5 nm [63,64]. This supports the assertion that these units may be an assembly of 4–5 nm diameter microfibrils [50], which is also consistent with the fact that surface proteoglycans, such as decorin, embrace multiple microfibrils (four or more) [48]. Therefore, it is intuitive to hypothesize that the larger D-periodical collagen fibrils may be formed by an assembly of slender (10–25 nm diameter), and perhaps more tightly linked, substructural units or microfibrillar bundles that are thinner than the parent D-periodical fibrils. The fact that microfibrillar extraction from collagen fibrils is nonexistent in the literature [50] also supports this perspective. This goes hand-in-hand with the perception that the thickening of developing collagen generally occurs from the lateral aggregation of structures measuring 10–20 nm in diameter, and not the ∼5 nm diameter that would be consistent with the lateral aggregation of single microfibrils.

Based on the above mentioned observations it becomes evident that gradual dissection of the hierarchically orchestrated supramolecular assemblies that constitute dentin collagen fibrils gradually reveals structural features that may impose critical limitations to the action of polymeric dental materials. Although collagen substructural units present as tightly assembled fibrillar entities at larger length scales, the evidence discussed above suggests that complete infiltration of collagen by monomer molecules of dental materials may be hampered by structural barriers formed by these internal substructures. As such, viscous polymers may envelop larger fibrils but may not penetrate them and surround the internal collagen features to the same degree. Hence, spots of poor impregnation/infiltration may result in sites more prone to hydrolytic degradation of the dentin–biomaterial interface. The interaction of adjacent substructural units within the D-periodical fibril will be further described in a subsequent section, along with the role of water in forming the so-called “cylinders of hydration” around collagen molecules, a phenomenon which we contend may be an important contributor to nanoleakage and the hydrolysis of interfaces between dentin and polymer-based dental materials.

#### 3.1.3 The microfibrillar level – orchestrated supramolecular assemblies forming thicker fibrillar entities

Thus far we have described the structural features of the nearly 100 nm diameter D-periodical dentin collagen fibrils and the (potentially transient) arrangement of its 10–25 nm diameter substructural units. As stated above, in this nanostructured hierarchical organization the 10–25 nm diameter substructural units are, in theory, an assembly of 4–5 nm diameter microfibrils. Microfibrils, in turn, represent the next hierarchical level of collagen type I (Fig. 1), and are formed by intertwined triple-helical collagen molecules, which each measure about 1.4 nm in diameter. Thus it may be noted that microfibrils represent the lowest hierarchical level of collagen type I at which a supramolecular structure is formed. Therefore, they may be considered the basic building blocks of collagen type I.

Numerous models have been proposed to describe the specific packing arrangement of collagen molecules that constitute a microfibril. XRD is the technique most frequently used to achieve the resolution necessary to understand the molecular packing within a collagen fibril. The majority of models proposed to date have been established on the basis of the Hodge and Petruska two-dimensional scheme [58]. The Hodge–Petruska scheme divides the collagen molecule into five units, which consist of five collagen monomers, M1–M5. All five units have a regular length, equivalent to 1 D, with the exception of M5, which in collagen type I has a length of approximately 0.46 D [52,62,67]. In an electron micrographic depiction of this model the so-called “gap zones” represent the region at the end of the short segment M5, whereas the lighter bands represent the areas where all five segments are present, and correspond to the “overlap zones”.

One popular model that has been proposed after the Hodge–Petruska scheme is the so-called Smith five stranded microfibril [59]. This three-dimensional model consists of a Hodge–Petruska monolayer of unlimited length and five molecules wide rolled up into a slender cylinder. Significant advancement was subsequently achieved when, in the late 1970s, Hulmes proposed his quasi-hexagonal packing model, which established that adjacent triple-helical molecules are arranged in a quasi-hexagonal fashion with respect to each other within the fibril [68]. From a more simplified perspective this means that the lateral organization of the five triple-helical molecules fit into a quasi-hexagonal outline from a cross-sectional view of one microfibril (Fig. 1). This quasi-hexagonal arrangement repeats itself along the fibril axis. This is important because, from a physiological standpoint, it is primarily how the collagen triple-helices are packed in a microfibril relative to one another that dictates the location and accessibility of its many biologically active ligand interaction sites [62]. Examples are crosslinking sites within the larger D-periodical fibril, binding sites for proteoglycans, non-collagenous proteins and proteases, such as matrix metalloproteinases (MMPs). These same sites, therefore, define fibril function, tissue organization, and various mechanisms for different diseases and homeostatic processes [49,69,70].

The most recent microfibril model, proposed by Orgel et al. [50], provides a more accurate characterization of microfibrils in collagen type I, by which all of the molecular segments within one D-period (where D is the 67 nm repeat), including the four molecular segments within the previously unresolved gap region, can be characterized [50]. Firstly, this investigation confirmed the model proposed by Hulmes, where each collagen molecule is arranged in a quasi-hexagonal fashion in the overlap region [68]. It also determined that this arrangement continues through the gap region, despite the absence of one collagen molecule at this location and the fact that each of the four molecular segments adopts a unique conformation within the gap zones [50]. In summary, Orgel et al. [50] defined microfibrils as a pentameric staggered arrangement of molecules that form a higherorder supramolecular structure with a right-handed supertwist. This model is also consistent with the assertion that each microfibril contains at least two or three inter-microfibrillar crosslinking sites and one intra-microfibrillar crosslinking site, which led to the hypothesis that collagen may be considered a rope network where the elements of the array transmit force to one another, whereas the microfibrillar elements maintain structural stability through the right-handed supertwist.

**Fig. 4:**
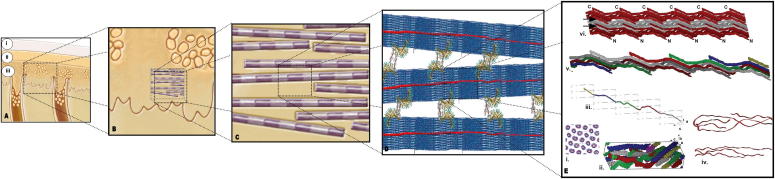
Schematic depiction of a hierarchical view of a hybrid layer and its constituents. (A-i) composite resin, (A-ii) adhesive layer, and (A-iii) monomer infiltrated dentin substrate. (A)–(C) represent increasing magnifications of the currently accepted concept of hybridization, where the D-periodical ∼100 nm diameter dentin collagen fibrils represent the ultimate structures to be impregnated and enveloped (adapted from Powers and Sakaguchi [143]). (D) Collagen fibrils (adapted from Gautieri et al. [144]) interconnected by the proteoglycan decorin (a monomeric representation of the dimeric model based on the available crystal structure of the protein core of decorin [145]). (E) Collagen microfibrillar organization and structure where the C-axis has been compressed for easier visualization (adapted from Orgel et al. [50]). (E-i) Model showing the quasihexagonal lateral packing of the molecular segments. (E-ii) Conformation of the D-staggered collagen segments within a single microfibril. (E-iii) The molecular path of a collagen molecule through successive microfibrils. (E-iv) Enlarged view of the N- (bottom) and C-telopeptide (top) regions of type I collagen. (E-v) Taking several 1D staggered collagen molecules from the collagen packing structure (single molecule shown in C) it is possible to represent the collagen microfibril. (E-vi) Three microfibrils are shown side by side to indicate the probable binding relationship between them.

The hybridization of dentin with polymeric bonding agents, originally proposed by Nakabayashi et al. [21], is a fascinating engineering concept that was conceived to enable the micromechanical retention of synthetic polymers on the complex biological substrate that is the tooth. This concept has since its advent represented the most important and far-reaching revolution in contemporary restorative dentistry. However, since the mechanistic concept of hybridization involves the infiltration of adhesive co-monomers (HEMA, triethyleneglycoldimethacrylate (TEGDMA) and, occasionally, urethane dimethacrylate(UDMA)) into demineralized type I collagen fibrils one may ascertain that this paradigm adopts a simplified perspective of the dentin substrate at the sub-fibrillar level, as schematically depicted in Fig. 4. We contend that these intricate supramolecular interactions in collagen offer a variety of structural, molecular and physical constraints against an improved interaction of the synthetic monomers with the dentin substrate on sub-micrometer scales.

The specific organization of the innerstructure of collagen fibrils has been extensively studied. These early reports pointed to important limitations on the ability of dental monomers to occupy the inner structure of dentin collagen, a perspective that has been poorly explored thus far. According to the classic model proposed by Hulmes [71], and more recently confirmed by Orgel et al. [50], the inter-molecular spacing in the lateral packing of collagen molecules within a microfibril (and hence within a collagen fibril), which is representative of the space putatively occupied by monomer molecules once they infiltrate the collagen structure, has been determined to range from 1.26 to 1.33 nm. If one considers the smallest monomer that is frequently used in dental polymers, i.e. TEGDMA, we may calculate the approximate length of the molecular backbone of a single extended monomer molecule based on the distance between its atomic bonds (C–C ≈ 120 pm, H–C ≈ 106 pm, and C–O ≈ 143 pm). The resulting monomer unit presents a length of roughly 2 nm per monomer molecule. Therefore, even from a simplified physical standpoint, complete infiltration of the adhesive material at an unrealistic single molecular level is limited by the space available between collagen molecules within a fibril, since the intermolecular space (1.26–1.33 nm) is unable to accommodate a *single* extended small monomer molecule (∼2 nm).

Furthermore, and maybe more importantly, it has been demonstrated that these intermolecular spaces are fully occupied by highly ordered and tightly bound water molecules which form multilayered cylinders of hydration around collagen molecules [57,72]. These so-called hydration shells guarantee, in turn, the specific register of the supramolecular structure [72] of collagen, and may be a critical factor leading to hydrolytic degradation of dental monomers on the nanoscale. This topic will be addressed in the following section.

#### 3.1.4 The molecular level – the role of water and correlative nanoscale hydrolytic phenomena

The molecular organization of collagen is an extremely broad science and we will limit our discussions to aspects relevant to understanding the interaction of polymeric dental materials with collagen molecules. Emphasis will be given to the role of water in the structure of collagen and the correlative hydrolytic phenomena on the nanoscale. As the literature on hydrolytic degradation of dental polymers is almost exclusively related to dental adhesives, our discussion will be primarily focused on previously reported mechanistic degradation of this specific range of materials. Nonetheless, we contend that similar mechanisms may take place in other polymers that establish ultrafine contact with the dentin collagenous network, such as adhesive cements, glass ionomers, liners, etc.

**Fig. 5:**
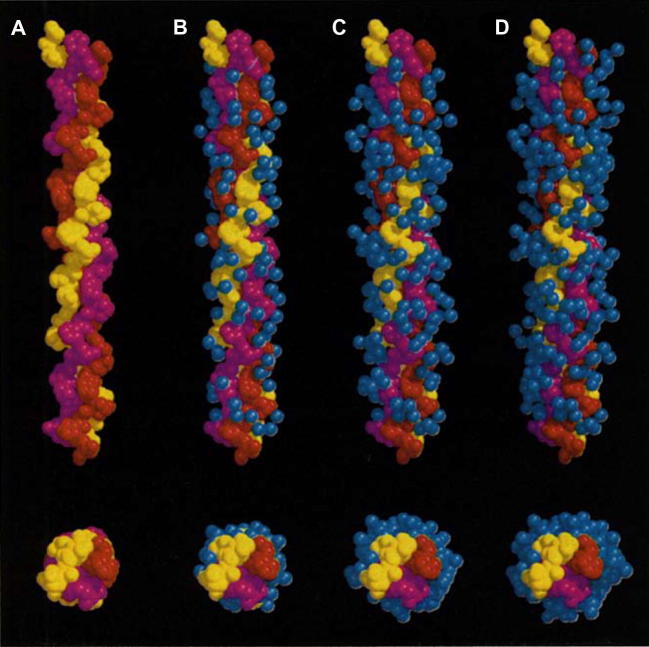
Spacefilling longitudinal (top) and cross-sectional (bottom) representations of the progressive hydration of the Gly-Ala peptide as seen in the crystal structure of the collagen molecule [72]. Each colour represents one peptide chain of the triple helix, whereas the water molecules are shown in blue. (A) A view of the molecule without water. Incorporation of the (B) first, (C) second and (D) third shells of water molecules. Water molecules are either directly hydrogen bonded to carbonyl, hydroxyl or even amide groups on the peptide surface or hydrogen bonded to the first or second shells of water molecules.

A single collagen molecule consists of three polypeptide chains composed of two α_1_ and one α_2_ sequences. The resulting molecular unit has a mass of about 285 kDa and is approximately 1.4 nm wide and 300 nm long. The basic triple-helical conformation consists of three closepacked supercoiled helices, which requires a glycine residue at every third position in the polypeptide chain [60]. This results in a (X–Y–Gly)*_n_* repeating pattern in which the X and Y positions are frequently occupied by proline and 4-hydroxyproline residues, respectively [62,72] (Fig. 1). The resulting polypeptide chain, therefore, assumes a left-handed helical conformation with about three residues per turn [60]. The assembled triple-helical molecular structure, on the other hand, is constituted by three parallel chains which wind around each other with a gentle right-handed supertwist to form the resulting molecular unit (Fig. 5).

It was recognized early on that water plays an important role in maintaining the conformation of native collagen molecules (for a review see Fraser and MacRae [73]). A variety of techniques and measurements, including nuclear magnetic resonance, dielectric measurements, water sorption and heat capacity, indicated that water is either tightly bound to specific sites on collagen chains or fills the spaces between the collagen molecules [74–79]. This idea initially stemmed from the assertion that the specific register of the three polypeptide chains in one collagen molecule relies on the presence of hydrogen bridges between Gly residues and the carboxyl oxygen (of an X (Pro) residue) from a neighbouring chain [57]. Nevertheless, an important study by Bella et al. [57] found that the lateral spacing between molecules is too long for direct hydrogen bonds to occur between specific residues. It was confirmed thereafter that these inter-chain and inter-molecular bonds are formed by inherent water molecules which form multispan hydrogen bonded bridges connecting neighbouring collagen molecules [57] (Fig. 5). Based on these investigations, there is general agreement that triple-helices are surrounded by a highly structured “cylinder of hydration”, and the effective diameter of these “cylinders” dictates the lateral separation in the macromolecular assemblies that form the resulting fibrillar units in collagen type I.

Concern regarding the hydrolytic degradation of polymeric restorations as a consequence of solvent uptake, which leads to a shortened service life, is a common finding in the dental literature [5,10,11,80]. There is consensus that the bonded interface remains the weakest area of toothcoloured restorations [10]. Statements such as “it is common to attempt to remove a restoration bonded to dentin only to find that the vibration of the cutting instrument causes it to fly off the bonded surface” are, unfortunately, still found in the literature [81]. There is agreement that the primary reason behind this less than desirable long-term performance relates to the hydrolytic nature of the monomer components of restorations bonded to the physiologically hydrated dentin structure [10,80].

The three most common monomers used in dentistry, namely 2,2-bis-GMA (bis-GMA), UDMA, and TEGDMA, as well as many other dimethacrylate monomers used as dental materials [82], retain chemical groups such as ester, urethane and hydroxyl groups and ether linkages that are susceptible to hydrolytic cleavage [80,83]. Not surprisingly, therefore, there is increasing evidence that hydrolytic degradative events occur at the dentin–polymer interface. Various patterns of degradation have been proposed, such as poorly impregnated collagen fibrils [84–88], elution of resin monomers [89–91] and degradation of resin components [84,85,92–95], all related to hydrolytic effects. Evidence of the rapid progression of this hydrolytic process (i.e. 6 months) has also been presented [96,97]. So the question that remains is: what exact mechanisms contribute to the hydrolytic degradation of polymeric restorations bonded to dentin?

We argue that a careful analysis of the highly complex and intrinsically hydrated hierarchical supramolecular assemblies that compose the dentin substrate offers a myriad of structural constraints that favour multispan hydrolytic events at the tooth–biomaterial interface on the sub-micrometer scale. For instance, the aforementioned high level of porosity and nano-voids at the fibril surface, where water molecules can be sequestered, leading to solvent uptake and elution [55]. It has also been suggested that a failure of water being completely removed from the collagen fibrils is possibly another reason why bond degradation may occur in dental adhesives [11]. To date there is no evidence that water in the intrafibrillar compartments of adhesive joints is completely replaced by resin, and this assertion may raise some scepticism. Here, on the basis of the vast literature concerning the molecular and nanoscale structure of collagen type I, we present evidence supporting the hypothesis that the complete impregnation of demineralized dentin collagen by dental monomers is limited by the physical constraints imposed by the intermolecular distance within collagen fibrils. Furthermore, we showed that these intermolecular spaces are intrinsically occupied by tightly bound water molecules. Thus the envelopment of collagen fibrils and their impregnation (if any) by hydrolysable monomers, irrespective of the bonding strategy, would favour the formation of an interface that is inherently susceptible to hydrolytic degradation on the nanoscale. Overall we argue that on the nanoscale and from a molecular standpoint the adhesive joint between dentin and dental polymers represents the antithesis of successful bonding.

A very interesting review recently published by Pashley et al. [5] offers a myriad of observations that go hand-in-hand with the theories proposed here. In this recent review the authors argued that a technique called ethanol wet-bonding could potentially overcome a number of limitations leading to the degradation of dentin–bonder interfaces. The ideas they proposed seem very reasonable to us. In this technique ethanol is used to chemically dehydrate acid etched, demineralized dentin matrices [98], which results in lateral shrinkage of the collagen fibrils, resulting in an increase in the size of the interfibrillar spaces and a reduction in the hydrophilicity of the collagen matrix. It has been demonstrated that ethanol wet-bonding does not compromise bond strength, at least when bis-GMA was utilized [54], which is an important advantage. Further, this technique seems to reduce nanoleakage and prevent the degradation of resin–dentin bonds [99]. In this same review the authors described a model whereby adhesives may interact with collagen in many different ways. Accordingly, there may be situations where there is too much water surrounding the fibrils, thus hampering polymerization of the adhesive. Another possibility is that excess water may prevent penetration of the adhesive into the fibrils, which is in agreement with our interpretation above. On the other hand, the authors propose that if there is a layer of ethanol surrounding the collagen the adhesive may dissolve and penetrate into the fibrils between the collagen molecules.

Our perspective of the ethanol wet-bonding is two-fold: first, the removal of interfibrillar water provides a tighter wrapping of the adhesive around the collagen, and the ethanol may well facilitate permeation of the adhesive into nanometer scale crevices on the collagen surface. Secondly, although dissolution may occur, our calculations suggest that the adhesive monomer molecules may still be unable to fit between the collagen peptides, more so if the intermolecular spaces have been significantly reduced after removing the water [5]. Important questions that then arise are: can water molecules really leave the innermost regions of collagen, as they are so tightly bound to it? If yes, what are the consequences of removing this important component on the mechanical motion of the interacting microfibrils in dissipating mechanical forces during tissue strain? Pashley et al. [5] suggested that if the interaction at the collagen–adhesive interface is ideal, collagen strained longitudinally would “stretch” the adhesive along with it, which is a reasonable theoretical assumption. However, the ability of the fibrils and the adhesive to elastically recoil once the intermolecular interactions have been disrupted by ethanol is uncertain. This may either function well or “mechanically degrade” the collagen at even faster rates, by slowly disrupting the intermolecular connections imparting the twisted organization to the collagen molecules and microfibrils, as shown in Fig. 1. Immediate conclusions that can be drawn from these interesting discussions are that: (1) there is an urgent need for more specific consideration of the collagen–adhesive interactions on the molecular and nanostructural scales; (2) the application of existing nanotechnologies that allow us to understand what is happening within collagen, such as XRD, should be encouraged in dental materials research.

## 4 The proteolytic degradation of dentin–biomaterial interfaces by MMPs – a mechanistic standpoint relative to the collagen packing arrangement

It has been shown that nanoleakage can occur in the absence of marginal gaps in biomaterial–dentin interfaces [100], which led to the conclusion that host-derived proteinases make an important contribution to the degradation of incompletely infiltrated collagen in bonded dentin [101], even when bacteria are not present [102,103]. The proteolytic degradation of dentin–biomaterial interfaces has gained significant attention in recent years, and nowadays represents a scientific field of its own. It is not the objective of this review to provide a thorough description of the activity of MMPs in dentin in relation to the mechanism of action of current adhesive systems as an excellent review on this aspect has recently been published, and we encourage the reader to refer to this publication [11] for further details. In this discussion we will focus on less commonly explored aspects relative to the specific molecular packing arrangement of collagen, which has been demonstrated to govern collagen proteolysis, particularly with respect to the action of MMPs.

Breschi et al. [10] and Visse and Nagase [104] described MMPs as a class of zinc- and calcium-dependent endopeptidases that are trapped within the mineralized dentin matrix during tooth development [5,105,106]. The release and subsequent activation of these proteinases during dentin bonding [101,107,108] are thought to be responsible for the degradation of collagen fibrils in incompletely infiltrated hybrid layers in aged bonded dentin [103,109–111]. An aspect that has received considerably less attention is that the molecular packing arrangement of collagen and its microfibrillar arrangement at the fibril surface is a critical aspect governing the exposure of binding sites available to collagenolytic macromolecules. MMPs with collagenolytic activity cleave the three α-peptide chains at a specific Gly–Ile/Leu bond [104] within the M4 unit of the collagen molecule (relative to the Hodge–Petruska scheme division of the collagen molecule into five units).

Most previous models of collagen proteolysis assumed that the fibrillar arrangement would freely accommodate MMPs to digest individual collagen monomers in the fibril [112,113]. However, the specific molecular interactions that enable binding of MMPs to the collagen surface, thus allowing subsequent collagenolysis, proved to be a much more complex mechanism. MMP1, the only member of the family for which such an interaction has been characterized thus far, has been used as a model to define how binding and collagenolysis might occur in the case of MMP degradation of collagen type I [49]. In this study the native environment of the MMP cleavage site on the type I fibril was determined from the XRD structure [50], and its effect on collagenolysis investigated.

**Fig. 6:**
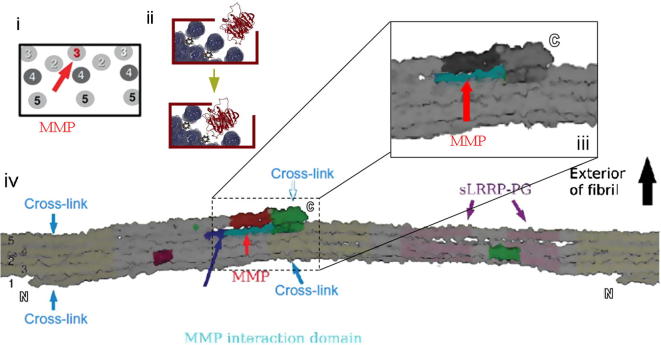
MMP-1 as a model for MMP driven collagenolysis. (i) The MMP cleavage site is buried in a narrow cleft at the fibril surface. (ii) MMP access to collagen degradation is thought to require C-telopeptide removal for full enzyme access (top), as illustrated in the bottom image. Removal may not have to result in cleavage, however, it may be possible for the enzyme to squeeze into the cleft if the C-terminal region is moved due to extrinsic events affecting the packing arrangement of the fibril, such as in cases of thermal motion, bending of the fibril or putatively demineralization with strong acids, such as the phosphoric acid conditioner of adhesive systems. (iii) Longitudinal view of collagen molecular packing illustrating the MMP cleavage site (cyan) partially covered by the C-telopeptide region (green). (iv) Higher magnification view of (iii). (Modified from Orgel et al. [62].)

The MMP molecule contains one catalytic domain, a flexible bridging component, which links the two “ends” of the molecule, and a substrate recognition C-terminal domain. The molecular model of the collagen–MMP interaction was developed based on the assertion that the active site of the MMP is a groove running across the surface of the catalytic domain [49] (Fig. 6). However, it has been determined that the active catalytic site of the MMP molecule is only 0.5 nm wide [114], and is therefore unable to accommodate the entire diameter of an intact collagen triple helix, which measures roughly 1.4 nm. Therefore, it has been hypothesised that MMPs may first bind to and then locally unwind the triple helix so that each peptide may fit into the active site binding groove, before hydrolysing the peptide bonds of each chain in succession [114]. However, the above mentioned study demonstrated that the entire collagen region where cleavage begins, namely the α2 chain within a narrow solvent-accessible cleft, is located in a region that is fully protected by the C-telopeptide (Fig 6). Therefore, access by MMPs to the cleavage site on the native (unchanged) collagen structure is greatly restricted [49].

It was proposed, therefore, that extrinsic factors may change the structure of collagen in order for collagenolysis to begin. The currently accepted hypothesis [49] is that when fibrils are damaged (such as in demineralization due to acid attack, i.e. in caries lesions or acid etching procedures) changes in the molecular arrangement, such as the breakage of cross-linkages at the C-terminus, may expose the catalytic binding site of the α2 chain, thus leading to an initial cleavage event. This initial cleavage, in turn, would facilitate interaction of the other chains with the MMP catalytic domain, thus triggering the collagenolytic process. Therefore, one may suggest that collagen fibrils retain crypto-biological features [62] that may intrinsically regulate disease progression and physiological events involving MMP degradation.

The above outcome supports the hypothesis that collagenases either disassociate [114] or target sections of the peptides that have been previously dissociated by external factors [115], such as acid etching procedures, and therefore are more vulnerable to attack by a proteolytic enzyme. It also supports the previously proposed theory that the acid etching used in bonding systems could facilitate the action of host-derived MMPs [101], triggering the collagenolytic and gelatinolytic identified activities within hybridized dentin. It should be noted, however, that although activation of MMPs occurs irrespective of its interaction with collagen, as previous studies have shown [105,116,117], it is likely that for actual collagenolytic activity a myriad of structural changes within the collagen packing arrangement may be required to allow specific binding of the catalytic domain of the MMP molecule to the targeted amino acid sequence in the α2 chain of the collagen molecule. This may also be valid for another type of collagen-degrading enzymes in dentin that has been recently identified, namely cysteine cathepsins [118,119]. Recent studies demonstrating the efficacy of cross-linking agents in increasing the longevity of resin–dentin bonds [120–125] support the hypothesis that the collagen structure plays a critical role in regulating MMP degradation. This is because cross-linkage of the laterally packed molecules makes it significantly more difficult for the MMP molecule to reach its α2 chain “target”. However, the fact that at least some of the cross-linkers (at least carbodiimide and proanthocyanidins) are also MMP inhibitors [126,127] should be taken into account.

As stated above, although it is not our intention to provide a thorough description of the activity of MMPs in dentin, we briefly reviewed recent experimental evidence for the action of some MMPs commonly found in dentin, particularly in relation to their collagenolytic activity relative to the use of self-etch and etch-and-rinse adhesive systems. Table 1 shows a chronological perspective of recent observations on the action of MMPs. An early study by Mazzoni et al. [128] suggested that acid etching with 10% phosphoric acid and 17% EDTA reduces the collagenolytic activity of endogenous dentin enzymes, whereas several etch-and-rinse adhesive systems appeared to re-activate the function of dentin MMPs. These observations go hand-in-hand with the results of Nishitani et al. [107], which showed that self-etch adhesives can also activate MMPs, whereas strong acids, such as phosphoric acid, are likely to denature MMPs given their extremely high acidity. A more recent study supported this perspective and provided TEM evidence that etch-and-rinse adhesives caused extensive degradation of the hybrid layer *in-vivo* [129]. De Munck et al. [130] used gelatine zymography to demonstrate that an etch-and-rinse adhesive system caused the release of MMP-2, whereas a self-etch adhesive yielded no release of endogenous enzymes at the adhesive–dentin interface. Conversely, studies by Lehmann et al. [147] suggested that a self-etch adhesive stimulated the secretion of MMP-2 and MMP9 from human odontoblast cells. Another study by Mazzoni et al. [148] used colorimetry and SEM/TEM immunohistochemistry and observed an increase in MMP-2 in the hybrid layer of dentin treated with an etch-and-rinse adhesive. Finally, a study using western blotting and immunofluorescence to compare self-etch and etch-and-rinse adhesive systems showed that the self-etch material caused a significantly higher expression of MMP-2 in human fibroblasts. This is just to name a few of the more recent studies. Our interpretation of Table 1 is that the adhesives of etch-and-rinse systems may stimulate MMP degradation of dentin collagen, although MMPs may be turned off by strong acids [128]. However, while mild acids of self-etch systems may not be strong enough to deactivate MMPs, the adhesive monomers may facilitate MMP activity even further. These observations are in good agreement with previous reviews [5,11]. A recent systematic review of Class V clinical data indicated that the failure rates of Class V bonded restorations follow the order: 1-step self-etch > 2-step etch-and-rinse > 2-step self-etch (aggressive) > 3-step etch-and-rinse > 2-step self-etch (mild and moderately aggressive). Liu et al. [11] suggested that the higher durability of the mild 2-step self-etch adhesives *in-vivo* may be attributed to the use of a more hydrophobic resin layer on top of the hydrophilic self-etch primer, which renders the interface less susceptible to water sorption, which we contend is a reasonable assumption. Further studies on the activity and specificity of MMPs in degrading bonded dentin collagen are certainly warranted.

## 5 Dentin proteoglycans – structural features relevant to understanding their interaction with dental polymers

**Fig. 7:**
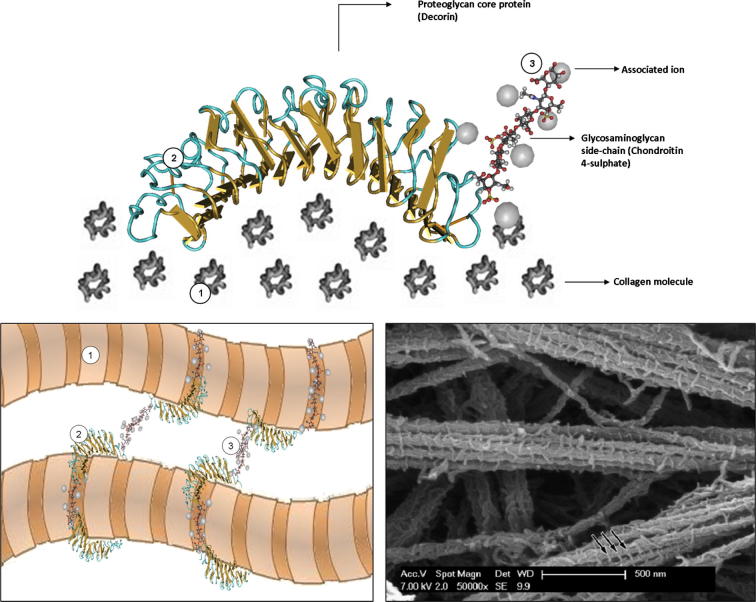
Schematic representation of proteoglycan attached to the collagen surface (not to scale). (A) Monomeric model of decorin based on the available crystal structure of the dimeric protein core [145]. The association of decorin with the collagen surface is based on a recently proposed model [48]. The glycosaminoglycan side-chain, based on the structure of chondroitin 4-sulfate [146] is positioned in a hypothetical region of the decorin protein core with associated ions. All molecular structures are available from the National Center for Biotechnology Information structure database (http://www.ncbi.nlm.nih.gov). (B) A not to scale schematic sketch of the interfibrillar supramolecular assemblies that interconnect collagen fibrils: (1) collagen fibril; (2) decorin protein core; (3) chondroitin 4-sulfate glycosaminoglycan. The known periodicity of these interfibrillar aggregates in register with the gap zones of collagen fibrils, present in most connective tissues, remains uncertain for mineralized tissues. (C) A high magnification image of a sample of acid-soluble collagen and decorin treated with cupromeronic blue, which reacts with glycosaminoglycans and demonstrates their assembly as interfibrillar co-aggregates (arrows) [127].

An improved understanding of the specific nanostructural interaction of proteoglycans with polymeric dental materials requires a critical reappraisal of proteoglycans as a biological entity and of their interaction with the collagen fibril surface [13,131] (Fig. 7). Decorin and biglycan, two members of the small leucine-rich repeat (SLRP) family, are the proteoglycans predominantly expressed in dentin [28]. It has been shown that proteoglycans retain a protein core that adopts a folded helical configuration stabilized by hydrogen bonds and hydrophobic and electrostatic interactions [132], which has also been suggested to bind to four or more collagen microfibrils via an array of hydrogen bonds (particularly decorin) (Fig. 7) [48].

Therefore, it must be highlighted that proteoglycans as a structural entity comprise a protein core (primarily decorin and biglycan in the case of dentin) generally associated with hydrophilic carbohydrate anionic glycosaminoglycan side-chains (Fig. 6). The glycosaminoglycans most frequently found in dentin are chondroitin 4-sulphate and a relatively lower content of chondroitin 6-sulphate [28], although dermatansulphate, hyaluronan and keratansulphate have also been reported [28]. These negatively charged carbohydrate side-chains interact with one another, thus forming interfibrillar bridges that absorb water, span the spaces between the fibrils and regulate the mechanical properties of the collagenous matrix [131,133–135].

In summary, it should be noted that proteoglycans form a complex supramolecular interfibrillar nanostructured scaffold that is primarily responsible for holding the collagenous network together. In restorative adhesive procedures, once the mineral (and the smear layer) is removed, this complex network becomes exposed and interaction of the adhesive matrix is not dependent only on micromechanical retention by the collagenous network but also to its interaction with the interfibrillar supramolecular assemblies formed by proteoglycans and glycosaminoglycans, and in the latter case this interaction occurs at a much finer scale. The participation of this organic scaffold in the micromechanical retention of dental adhesives has been previously demonstrated. However, while two studies demonstrated reduced bond strength of dentin lacking glycosaminoglycans and proteoglycans [136,137], other investigators showed an increase in bond strength after glycosaminoglycan digestion [138]. The studies showing a reduction in bond strength used a three-step adhesive system [136,137], whereas the research showing an average increase of 92% in bond strength used a two-step etch-and-rinse system [138], while the bond strengths of specimens treated with a three-step adhesive showed a much lower increase of 28%. We are unable to explain these discrepancies, and more research may be necessary to understand this phenomenon. Regardless of the outcomes resulting from the removal of glycosaminoglycans, care must be exercised in drawing conclusions from these cited reports. In the long-term glycosaminoglycans and proteoglycans may also represent sites where the diffusion of water molecules is facilitated, given the highly hydrophilic character of the resulting molecular assembly. This may facilitate hydrolytic degradation of the bonding monomers and promote an environment that is more prone to leakage. Furthermore, the inhibitory action of proteoglycans on MMPs in dentin [139] should be carefully studied.

An important aspect that must be considered in this regard is the ability of viscous monomers to hermetically surround the highly intricate non-collagenous organic network. Fig. 7 offers a not to scale depiction of the proteoglycan structure, illustrating the complexity of the super-coiled spring-like protein core and interaction of the tape-like co-aggregates of antiparallel glycosaminoglycan strings bridging adjacent collagen fibrils. The participation of water molecules and other ionic media is crucial to ensure that full extension of the proteoglycan–glycosaminoglycan complex is achieved [131,133]. This in turn guarantees separation between the collagen fibrils and promotes a significant degree of osmotic pressure in the organic matrix [131,133], which plays an important role in the elastic and visco-elastic properties of the dentin substrate. Furthermore, experiments promoting the aggregation of glycosaminoglycans on collagen type I have demonstrated that the glycosaminoglycan carbohydrate filaments sit on and envelop the collagen surface [140], thus representing another potential “barrier” to the penetration of bonding monomers into both the intra- and extra-fibrillar compartments of the collagen fibrils and envelop them. These observations may offer further insights into the formation of nanometer voids within the hybrid layer [141], which in the long-term facilitate diffusion of water molecules, thus promoting hydrolysis and leading to nanoleakage. Future studies on the ability of viscous polymers to penetrate and interact with highly hydrated proteoglycan–glycosaminoglycan complexes, particularly at the nanoscale and molecular levels, are encouraged to provide insights into the topics raised in this review.

## 6 Conclusions

In summary, we have reviewed recent evidence supporting the hypothesis that the dentin matrix is composed of highly orchestrated protein assemblies that present extremely complex organizational features at the nanoscale and molecular levels. We argue that these complex organic structures undergo intricate interactions with polymeric restorative dental materials. Nanometer scale topographical features have been suggested to potentially hamper the hermetic encapsulation of dentin collagen fibrils. At the higher level of intrafibrillar complexity collagen substructural units have been proposed as preventing complete infiltration of the D-periodical collagen fibrils by dental monomers, a mechanistic conjecture that is further affected by the complexity of the subsequent hierarchical level, namely collagen microfibrils. Accordingly, the microfibrilar arrangement of collagen type I, which results from the pentameric staggered organization of the molecules in a higher order supramolecular supertwisted nanostructure, have been shown to result in collagen with an intermolecular spacing of ∼1.3 nm, which is too small to accommodate one single extended monomer molecule of common dental monomers (∼2 nm). Furthermore, these intermolecular spaces have been shown to be fully occupied by tightly bound water molecules, which we contend may be a critical contributor to the hydrolytic degradation of dental polymers. Additionally, we have reviewed recent observations on the action of MMPs in the light of their molecular interactions with collagen type I and offer insights into the contribution of proteoglycans and glycosaminoglycans to the hydrolytic events observed at dentin–polymer interfaces. The high complexity of these inherently hydrated supramolecular nano-aggregates suggests that optimal interaction between the current hydrolysable polymers and dentin remains to be achieved. This may contribute to the formation of an environment that is more prone to degradation, thus reducing the long-term prospects for current tooth coloured restorations.

## Figures and Tables

**Table 1:** Recent observations of the action of MMPs relative to different etch-and-rinse and self-etch adhesive systems.

Authors	MMP	Adhesive system		Method	Observations
Type	Product
Nishitani et al. [107]	Collagenolytic activity	Self-etch	Clearfil Tri-S Bond (Kuraray Medical, Tokyo, Japan)	Fluorescein-labelled gelatine	Sufficiently acidic to activate gelatinolytic and collagenolytic activities in mineralized dentin powder
Clearfil SE Bond primer (Kuraray Medical)
G-Bond (GC Corp., Tokyo, Japan)
Mazzoni et al. [128]	Collagenolytic activity	Etch-and-rinse	Adper Prompt L-Pop (3 M-ESPE, St Paul, MN)	Fluorescein-labelled gelatine and TEM	Etch-and-rinse adhesives reactivate MMP while acid etching alone reduces collagenolytic activity
One-Step (Bisco Inc., Schaumburg, IL)
Single Bond (3 M ESPE, St. Paul, MN)
Excite (Ivoclar-Vivadent, Schaan, Liechtenstein)
OptiBond Solo Plus (Sybron-Kerr, Orange, CA)
Brackett et al. [129]	Collagenolytic activity	Etch-and-rinse	Prime&Bond NT (DentsplyDeTrey, Konstanz, Germany)	TEM	Etch-and-rinse system caused extensive degradation of HL invivo
De Munck et al. [130]	MMP-2 MMP-9	Etch-and-rinse	OptiBond FL	Gelatinzymography	Release of MMP-2
Self-etch	Clearfill SE Bond (Kuraray America, New York, NY)	Gelatinzymography	No release of enzymes
Lehmann et al. [147]	MMP-2 MMP-9	Self-etch	Xeno III (DentsplyDeTrey, Konstanz, Germany)	Immunochemistry and zymography	Stimulates secretion of MMPs from odontoblasts
Mazzoni et al. [148]	MMP-2	Etch-and-rinse	AdperScotchbond 1 XT (3 M ESPE, St. Paul, MN)	Colorimetry and SEM/TEM immunohistochemistry	Application increased the presence of MMP-2 in HL
Orsini et al. [116]	MMP-2	Self-etch	TechBond (Isasan, RovelloPorro, Italy)	Western blot and immunofluorescence	Significantly higher MMP-2 expression in human fibroblasts
Etch-and-rinse	Optibond Solo	Western blot and immunofluorescence	

## Appendix A Figures with essential colour discrimination

Certain figures in this article, particularly Figures 1–7, are difficult to interpret in black and white. The full colour images can be found in the on-line version, at http://dx.doi.org/10.1016/j.actbio.2012.02.022.

## References

[1] Beltran-Aguilar ED, Barker LK, Canto MT, Dye BA, Gooch BF, Griffin SO, et al. Surveillance for dental caries, dental sealants, tooth retention, edentulism, and enamel fluorosis –United States, 1988–1994 and 1999–2002. Surveillance Summaries 54: MMWR. Atlanta, GA: Centers for Disease Control and, Prevention; 2005. p. 1–13. 16121123

[2] NHEA. National health expenditure projections 2009–2019. Washington, DC: US Department of Health and Human Services; 2006.

[3] Hannig M., Hannig C. (2010). Nanomaterials in preventive dentistry. Nat Nanotechnol.

[4] Ferracane J.L. (2011). Resin composite– state of the art. Dent Mater.

[5] Pashley D.H., Tay F.R., Breschi L., Tjaderhane L., Carvalho R.M., Carrilho M. (2011). State of the art etch-and-rinse adhesives. Dent Mater.

[6] Tay F.R., Pashley D.H. (2002). Dental adhesives of the future. J Adhes Dent.

[7] Vaderhobli R.M. (2011). Advances in dental materials. Dent Clin Nor Am.

[8] Manhart J., Chen H., Hamm G., Hickel R. (2004). Buonocore Memorial Lecture. Review of the clinical survival of direct and indirect restorations in posterior teeth of the permanent dentition. Oper Dent.

[9] Uskoković V., Bertassoni L.E. (2010). Nanotechnology in dental sciences: moving towards a finer way of doing dentistry. Materials.

[10] Breschi L., Mazzoni A., Ruggeri A., Cadenaro M., Di Lenarda R., De Stefano Dorigo E. (2008). Dental adhesion review: aging and stability of the bonded interface. Dent Mater.

[11] Liu Y., Tjaderhane L., Breschi L., Mazzoni A., Li N., Mao J. (2011). Limitations in bonding to dentin and experimental strategies to prevent bond degradation. J Dent Res.

[12] Marshall G.W., Marshall S.J., Kinney J.H., Balooch M. (1997). The dentin substrate: structure and properties related to bonding. J Dent.

[13] Bertassoni L.E., Stankoska K., Swain M.V. (2012). Insights into the structure and composition of the peritubular dentin organic matrix and the lamina limitans. Micron.

[14] Katz E.P., Li S.T. (1973). Structure and function of bone collagen fibrils. J Mol Biol.

[15] Katz EP, Wachtel E, Yamauchi M, Mechanic GL. The structure of mineralized collagen fibrils. Connect Tissue Res 1989;21:149–54 [discussion 55–8]. 10.3109/03008208909050005 2605938

[16] Landis W.J. (1996). Mineral characterization in calcifying tissues: atomic, molecular and macromolecular perspectives. Connect Tissue Res.

[17] Balooch M., Habelitz S., Kinney J.H., Marshall S.J., Marshall G.W. (2008). Mechanical properties of mineralized collagen fibrils as influenced by demineralization. J Struct Biol.

[18] Landis W.J., Hodgens K.J., Song M.J., Arena J., Kiyonaga S., Marko M. (1996). Mineralization of collagen may occur on fibril surfaces: evidence from conventional and high-voltage electron microscopy and three-dimensional imaging. J Struct Biol.

[19] Bertolotti R.L. (1991). Total etch–the rational dentin bonding protocol. J Esthet Dent.

[20] Perdigao J., Lambrechts P., van Meerbeek B., Tome A.R., Vanherle G., Lopes A.B. (1996). Morphological field emission-SEM study of the effect of six phosphoric acid etching agents on human dentin. Dent Mater.

[21] Nakabayashi N., Kojima K., Masuhara E. (1982). The promotion of adhesion by the infiltration of monomers into tooth substrates. J Biomed Mater Res.

[22] Sano H., Shono T., Takatsu T., Hosoda H. (1994). Microporous dentin zone beneath resin-impregnated layer. Oper Dent.

[23] Van Meerbeek B., Dhem A., Goret-Nicaise M., Braem M., Lambrechts P., VanHerle G. (1993). Comparative SEM and TEM examination of the ultrastructure of the resin–dentin interdiffusion zone. J Dent Res.

[24] Hashimoto M., Ohno H., Sano H., Kaga M., Oguchi H. (2003). In vitro degradation of resin–dentin bonds analyzed by microtensile bond test, scanning and transmission electron microscopy. Biomaterials.

[25] Sano H. (2006). Microtensile testing, nanoleakage, and biodegradation of resin–dentin bonds. J Dent Res.

[26] Tay F.R., Hashimoto M., Pashley D.H., Peters M.C., Lai S.C., Yiu C.K. (2003). Aging affects two modes of nanoleakage expression in bonded dentin. J Dent Res.

[27] Sano H., Takatsu T., Ciucchi B., Horner J.A., Matthews W.G., Pashley D.H. (1995). Nanoleakage: leakage within the hybrid layer. Oper Dent.

[28] Goldberg M., Takagi M. (1993). Dentine proteoglycans: composition, ultrastructure and functions. Histochem J.

[29] Linde A., Robins S.P. (1988). Quantitative assessment of collagen crosslinks in dissected predentin and dentin. Coll Relat Res.

[30] Boskey A.L. (1991). The role of extracellular matrix components in dentin mineralization. Crit Rev Oral Biol Med.

[31] Kinney J.H., Pople J.A., Marshall G.W., Marshall S.J. (2001). Collagen orientation and crystallite size in human dentin: a small angle X-ray scattering study. Calcif Tissue Int.

[32] Lin C.P., Douglas W.H., Erlandsen S.L. (1993). Scanning electron microscopy of type I collagen at the dentin–enamel junction of human teeth. J Histochem Cytochem.

[33] Perdigao J., Thompson J.Y., Toledano M., Osorio R. (1999). An ultra-morphological characterization of collagen-depleted etched dentin. Am J Dent.

[34] Breschi L., Gobbi P., Lopes M., Prati C., Falconi M., Teti G. (2003). Immunocytochemical analysis of dentin: a double-labeling technique. J Biomed Mater Res A.

[35] Breschi L., Perdigao J., Gobbi P., Mazzotti G., Falconi M., Lopes M. (2003). Immunocytochemical identification of type I collagen in acid-etched dentin. J Biomed Mater Res A.

[36] Nalla R.K., Porter A.E., Daraio C., Minor A.M., Radmilovic V., Stach E.A. (2005). Ultrastructural examination of dentin using focused ion-beam cross-sectioning and transmission electron microscopy. Micron.

[37] Habelitz S., Balooch M., Marshall S.J., Balooch G., Marshall G.W. (2002). In situ atomic force microscopy of partially demineralized human dentin collagen fibrils. J Struct Biol.

[38] Marshall G.W., Balooch M., Kinney J.H., Marshall S.J. (1995). Atomic force microscopy of conditioning agents on dentin. J Biomed Mater Res.

[39] Marshall G.W., Balooch M., Tench R.J., Kinney J.H., Marshall S.J. (1993). Atomic force microscopy of acid effects on dentin. Dent Mater.

[40] Bertassoni L.E., Habelitz S., Pugach M., Soares P.C., Marshall S.J., Marshall G.W. (2010). Evaluation of surface structural and mechanical changes following remineralization of dentin. Scanning.

[41] Vargas M.A., Cobb D.S., Denehy G.E. (1997). Interfacial micromorphology and shear bond strength of single-bottle primer/adhesives. Dent Mater.

[42] Garberoglio R., Brannstrom M. (1976). Scanning electron microscopic investigation of human dentinal tubules. Arch Oral Biol.

[43] Pashley D.H. (1991). Clinical correlations of dentin structure and function. J Prosthet Dent.

[44] Avery J.K. (1988). Oral development and histology.

[45] Fratzl P. (2008). Collagen: strucutre and mechanics.

[46] Orgel JP., San Antonio JD., Antipova O. (2011). Molecular and structural mapping of collagen fibril interactions. Connect Tissue Res.

[47] Orgel JP., Antipova O., Sagi I., Bitler A., Qiu D., Wang R. (2011). Collagen fibril surface displays a constellation of sites capable of promoting fibril assembly, stability, and hemostasis. Connect Tissue Res.

[48] Orgel J.P., Eid A., Antipova O., Bella J., Scott J.E. (2009). Decorin core protein (decoron) shape complements collagen fibril surface structure and mediates its binding. PLoS One.

[49] Perumal S., Antipova O., Orgel J.P. (2008). Collagen fibril architecture, domain organization, and triple-helical conformation govern its proteolysis. Proc Natl Acad Sci USA.

[50] Orgel J.P., Irving T.C., Miller A., Wess T.J. (2006). Microfibrillar structure of type I collagen in situ. Proc Natl Acad Sci USA.

[51] Orgel J.P., Miller A., Irving T.C., Fischetti R.F., Hammersley A.P., Wess T.J. (2001). The in situ supermolecular structure of type I collagen. Structure.

[52] Orgel J.P., Wess T.J., Miller A. (2000). The in situ conformation and axial location of the intermolecular cross-linked non-helical telopeptides of type I collagen. Structure.

[53] Raspanti M., Congiu T., Guizzardi S. (2001). Tapping-mode atomic force microscopy in fluid of hydrated extracellular matrix. Matrix Biol.

[54] Raspanti M., Ottani V., Ruggeri A. (1989). Different architectures of the collagen fibril: morphological aspects and functional implications. Int J Biol Macromol.

[55] Pace RJ, Datyner A. Model of sorption of simple molecules in polymers. J Polym Sci Polym Phys 1980;18.

[56] Pioch T., Staehle H.J., Duschner H., Garcia-Godoy F. (2001). Nanoleakage at the composite–dentin interface. a review. Am J Dent.

[57] Bella J, Eaton M, Brodsky B, Berman HM. Crystal and molecular structure of a collagen-like peptide at 1.9 Å resolution. Science 1994;266:75–81. 10.1126/science.7695699 7695699

[58] Petruska JA, Hodge AJ. A subunit model for the tropocollagenmacromolecule. Proc Natl Acad Sci USA 1964;51:871–6. 10.1073/pnas.51.5.871 PMC300176 14173005

[59] Smith J.W. (1968). Molecular pattern in native collagen. Nature.

[60] Voet D, Judith GV, Charlotte WP. Biochemistry. 3rd ed. Hoboken, NJ: Wiley; 2008.

[61] Ottani V., Raspanti M., Ruggeri A. (2001). Collagen structure and functional implications. Micron.

[62] Orgel J.P., San Antonio J.D., Antipova O. (2011). Molecular and structural mapping of collagen fibril interactions. Connect Tissue Res.

[63] Scott J.E. (1990). Proteoglycan: collagen interactions and subfibrillar structure in collagen fibrils. Implications in the development and ageing of connective tissues. J Anat.

[64] Yamamoto S., Hashizume H., Hitomi J., Shigeno M., Sawaguchi S., Abe H. (2000). The subfibrillar arrangement of corneal and scleral collagen fibrils as revealed by scanning electron and atomic force microscopy. Arch Histol Cytol.

[65] Raspanti M., Viola M., Sonaggere M., Tira M.E., Tenni R. (2007). Collagen fibril structure is affected by collagen concentration and decorin. Biomacromolecules.

[66] Silver F.H., Langley K.H., Trelstad R.L. (1979). Type I collagen fibrillogenesis: initiation via reversible linear and lateral growth steps. Biopolymers.

[67] Antipova O., Orgel J.P. (2010). In situ D-periodic molecular structure of type II collagen. J Biol Chem.

[68] Hulmes D.J., Miller A. (1979). Quasi-hexagonal molecular packing in collagen fibrils. Nature.

[69] Sweeney S.M., Orgel J.P., Fertala A., McAuliffe J.D., Turner K.R., Di Lullo G.A. (2008). Candidate cell and matrix interaction domains on the collagen fibril, the predominant protein of vertebrates. J Biol Chem.

[70] Twardowski T., Fertala A., Orgel J.P., San Antonio J.D. (2007). Type I collagen and collagen mimetics as angiogenesis promoting superpolymers. Curr Pharm Des.

[71] Hulmes D.J., Wess T.J., Prockop D.J., Fratzl P. (1995). Radial packing, order, and disorder in collagen fibrils. Biophys J.

[72] Bella J., Brodsky B., Berman H.M. (1995). Hydration structure of a collagen peptide. Structure.

[73] Fraser R.D.B., MacRae T.P. (1973). Conformation in fibrous proteins.

[74] Berendsen H.J.C., Migchelsen C. (1965). Hydration structure of fibrous macromolecules. Ann NY Acad Sci.

[75] Grigera J.R., Berendsen H.J.C. (1979). The molecular details of collagen hydration. Biopolymers.

[76] Nomura S., Hiltner A., Lando J.B., Baer E. (1977). Interaction of water with native collagen. Biopolymers.

[77] Cusack S., Lees S. (1984). Variation of longitudinal acoustic velocity at gigahertz frequencies with water content in rat-tail tendon fibers. Biopolymers.

[78] Peto S., Gillis P., Henri V.P. (1990). Structure and dynamics of water in tendon from NMR relaxation measurements. Biophys J.

[79] Hoeve C.A., Lue P.C. (1974). The structure of water absorbed in collagen. I. The dielectric properties. Biopolymers.

[80] Ferracane J.L. (2006). Hygroscopic and hydrolytic effects in dental polymer networks. Dent Mater.

[81] Christensen G.J. (2005). Bonding to dentin and enamel where does it stand in 2005?. J Am Dent Assoc.

[82] Peutzfeldt A. (1997). Resin composites in dentistry: the monomer systems. Eur J Oral Sci.

[83] Venz S., Dickens B. (1991). NIR-spectroscopic investigation of water sorption characteristics of dental resins and composites. J Biomed Mater Res.

[84] Santerre J.P., Shajii L., Tsang H. (1999). Biodegradation of commercial dental composites by cholesterol esterase. J Dent Res.

[85] Munksgaard E.C., Freund M. (1990). Enzymatic hydrolysis of (di)methacrylates and their polymers. Scand J Dent Res.

[86] Bean T.A., Zhuang W.C., Tong P.Y., Eick J.D., Yourtee D.M. (1994). Effect of esterase on methacrylates and methacrylate polymers in an enzyme simulator for biodurability and biocompatibility testing. J Biomed Mater Res.

[87] Santerre J.P., Shajii L., Leung B.W. (2001). Relation of dental composite formulations to their degradation and the release of hydrolyzed polymeric-resin-derived products. Crit Rev Oral Biol Med.

[88] Finer Y., Santerre J.P. (2004). The influence of resin chemistry on a dental composite’s biodegradation. J Biomed Mater Res A.

[89] Willershausen B., Callaway A., Ernst C.P., Stender E. (1999). The influence of oral bacteria on the surfaces of resin-based dental restorative materials–an in vitro study. Int Dent J.

[90] Pearson G.J. (1979). Long term water sorption and solubility of composite filling materials. J Dent.

[91] Toledano M., Osorio R., Osorio E., Fuentes V., Prati C., Garcia-Godoy F. (2003). Sorption and solubility of resin-based restorative dental materials. J Dent.

[92] Oysaed H., Ruyter I.E. (1986). Water sorption and filler characteristics of composites for use in posterior teeth. J Dent Res.

[93] Musanje L., Shu M., Darvell B.W. (2001). Water sorption and mechanical behaviour of cosmetic direct restorative materials in artificial saliva. Dent Mater.

[94] Ferracane J.L. (1994). Elution of leachable components from composites. J Oral Rehabil.

[95] Braden M., Causton E.E., Clarke R.L. (1976). Diffusion of water in composite filling materials. J Dent Res.

[96] Milleding P., Karlsson S., Nyborg L. (2003). On the surface elemental composition of non-corroded and corroded dental ceramic materials in vitro. J Mater Sci Mater Med.

[97] Ferracane J.L. (1997). Water sorption and solubility of experimental dental composites. Polymer Preprints.

[98] Nishitani Y., Yoshiyama M., Donnelly A.M., Agee K.A., Sword J., Tay F.R. (2006). Effects of resin hydrophilicity on dentin bond strength. J Dent Res.

[99] Sadek F.T., Castellan C.S., Braga R.R., Mai S., Tjaderhane L., Pashley D.H. (2010). One-year stability of resin–dentin bonds created with a hydrophobic ethanol-wet bonding technique. Dent Mater.

[100] Ferrari M., Tay F.R. (2003). Technique sensitivity in bonding to vital, acid-etched dentin. Oper Dent.

[101] Pashley D.H., Tay F.R., Yiu C., Hashimoto M., Breschi L., Carvalho R.M. (2004). Collagen degradation by host-derived enzymes during aging. J Dent Res.

[102] Carrilho M.R., Geraldeli S., Tay F., de Goes M.F., Carvalho R.M., Tjaderhane L. (2007). In vivo preservation of the hybrid layer by chlorhexidine. J Dent Res.

[103] Garcia-Godoy F., Tay F.R., Pashley D.H., Feilzer A., Tjaderhane L., Pashley E.L. (2007). Degradation of resin-bonded human dentin after 3 years of storage. Am J Dent.

[104] Visse R., Nagase H. (2003). Matrix metalloproteinases and tissue inhibitors of metalloproteinases: structure, function, and biochemistry. Circ Res.

[105] Tjaderhane L., Larjava H., Sorsa T., Uitto V.J., Larmas M., Salo T. (1998). The activation and function of host matrix metalloproteinases in dentin matrix breakdown in caries lesions. J Dent Res.

[106] van Strijp A.J., Jansen D.C., DeGroot J., ten Cate J.M., Everts V. (2003). Host-derived proteinases and degradation of dentine collagen in situ. Caries Res.

[107] Nishitani Y., Yoshiyama M., Wadgaonkar B., Breschi L., Mannello F., Mazzoni A. (2006). Activation of gelatinolytic/collagenolytic activity in dentin by self-etching adhesives. Eur J Oral Sci.

[108] Tay F.R., Pashley D.H., Loushine R.J., Weller R.N., Monticelli F., Osorio R. (2006). Self-etching adhesives increase collagenolytic activity in radicular dentin. J Endod.

[109] Brackett W.W., Ito S., Tay F.R., Haisch L.D., Pashley D.H. (2005). Microtensile dentin bond strength of self-etching resins: effect of a hydrophobic layer. Oper Dent.

[110] De Munck J., Van Meerbeek B., Yoshida Y., Inoue S., Vargas M., Suzuki K. (2003). Four-year water degradation of total-etch adhesives bonded to dentin. J Dent Res.

[111] Armstrong S.R., Vargas M.A., Chung I., Pashley D.H., Campbell J.A., Laffoon J.E. (2004). Resin–dentin interfacial ultrastructure and microtensile dentin bond strength after five-year water storage. Oper Dent.

[112] Siljander P., Lassila R. (1999). Studies of adhesion-dependent platelet activation: distinct roles for different participating receptors can be dissociated by proteolysis of collagen. Arterioscler Thromb Vasc Biol.

[113] Zaman M.H., Trapani L.M., Sieminski A.L., Mackellar D., Gong H., Kamm R.D. (2006). Migration of tumor cells in 3D matrices is governed by matrix stiffness along with cell–matrix adhesion and proteolysis. Proc Natl Acad Sci USA.

[114] Chung L., Dinakarpandian D., Yoshida N., Lauer-Fields J.L., Fields G.B., Visse R. (2004). Collagenase unwinds triple-helical collagen prior to peptide bond hydrolysis. Embo J.

[115] Stultz C.M. (2002). Localized unfolding of collagen explains collagenase cleavage near imino-poor sites. J Mol Biol.

[116] Orsini G., Mazzoni A., Orciani M., Putignano A., Procaccini M., Falconi M. (2011). Matrix metalloproteinase-2 expression induced by two different adhesive systems on human pulp fibroblasts. J Endod.

[117] Davis G.E. (1991). Identification of an abundant latent 94-kDa gelatin-degrading metalloprotease in human saliva which is activated by acid exposure: implications for a role in digestion of collagenous proteins. Arch Biochem Biophys.

[118] Tersariol I.L., Geraldeli S., Minciotti C.L., Nascimento F.D., Paakkonen V., Martins M.T. (2010). Cysteine cathepsins in human dentin–pulp complex. J Endod.

[119] Nascimento F.D., Minciotti C.L., Geraldeli S., Carrilho M.R., Pashley D.H., Tay F.R. (2011). Cysteine cathepsins in human carious dentin. J Dent Res.

[120] Al-Ammar A., Drummond J.L., Bedran-Russo A.K. (2009). The use of collagen cross-linking agents to enhance dentin bond strength. J Biomed Mater Res B Appl Biomater.

[121] Macedo G.V., Yamauchi M., Bedran-Russo A.K. (2009). Effects of chemical cross-linkers on caries-affected dentin bonding. J Dent Res.

[122] Bedran-Russo A.K., Yoo K.J., Ema K.C., Pashley D.H. (2009). Mechanical properties of tannic-acid-treated dentin matrix. J Dent Res.

[123] Bedran-Russo A.K., Vidal C.M., Dos Santos P.H., Castellan C.S. (2010). Long-term effect of carbodiimide on dentin matrix and resin–dentin bonds. J Biomed Mater Res B Appl Biomater.

[124] Castellan C.S., Pereira P.N., Grande R.H., Bedran-Russo A.K. (2010). Mechanical characterization of proanthocyanidin–dentin matrix interaction. Dent Mater.

[125] Castellan C.S., Pereira P.N., Viana G., Chen S.N., Pauli G.F., Bedran-Russo A.K. (2010). Solubility study of phytochemical cross-linking agents on dentin stiffness. J Dent.

[126] Tezvergil-Mutluay A., Mutluay M.M., Agee K.A., Seseogullari-Dirihan R., Hoshika T., Cadenaro M. (2012). Carbodiimidecross-linking inactivates soluble and matrix-bound MMPs, in vitro. J Dent Res.

[127] Cova A., Breschi L., Nato F., Ruggeri A., Carrilho M., Tjaderhane L. (2011). Effect of UVA-activated riboflavin on dentin bonding. J Dent Res.

[128] Mazzoni A., Pashley D.H., Nishitani Y., Breschi L., Mannello F., Tjaderhane L. (2006). Reactivation of inactivated endogenous proteolytic activities in phosphoric acid-etched dentine by etch-and-rinse adhesives. Biomaterials.

[129] Brackett M.G., Tay F.R., Brackett W.W., Dib A., Dipp F.A., Mai S. (2009). In vivo chlorhexidine stabilization of hybrid layers of an acetone-based dentin adhesive. Oper Dent.

[130] De Munck J., Van den Steen P.E., Mine A., Van Landuyt K.L., Poitevin A., Opdenakker G. (2009). Inhibition of enzymatic degradation of adhesive–dentin interfaces. J Dent Res.

[131] Scott J.E., Thomlinson A.M. (1998). The structure of interfibrillar proteoglycan bridges (shape modules) in extracellular matrix of fibrous connective tissues and their stability in various chemical environments. J Anat.

[132] Bella J., Hindle K.L., McEwan P.A., Lovell S.C. (2008). The leucine-rich repeat structure. Cell Mol Life Sci.

[133] Scott J.E. (1992). Supramolecular organization of extracellular matrix glycosaminoglycans, in vitro and in the tissues. Faseb J.

[134] Scott J.E. (2003). Elasticity in extracellular matrix ‘shape modules’ of tendon, cartilage, etc. A sliding proteoglycan-filament model. J Physiol.

[135] Scott J.E. (2008). Cartilage is held together by elastic glycan strings. Physiological and pathological implications. Biorheology.

[136] Bedran-Russo A.K., Pereira P.N., Duarte W.R., Okuyama K., Yamauchi M. (2008). Removal of dentin matrix proteoglycans by trypsin digestion and its effect on dentin bonding. J Biomed Mater Res B Appl Biomater.

[137] Pereira P.N., Bedran-de-Castro A.K., Duarte W.R., Yamauchi M. (2007). Removal of noncollagenous components affects dentin bonding. J Biomed Mater Res B Appl Biomater.

[138] Mazzoni A., Pashley D.H., Ruggeri A., Vita F., Falconi M., Di Lenarda R. (2008). Adhesion to chondroitinase ABC treated dentin. J Biomed Mater Res B Appl Biomater.

[139] Hadler-Olsen E., Fadnes B., Sylte I., Uhlin-Hansen L., Winberg J.O. (2011). Regulation of matrix metalloproteinase activity in health and disease. FEBS J.

[140] Raspanti M., Viola M., Forlino A., Tenni R., Gruppi C., Tira M.E. (2008). Glycosaminoglycans show a specific periodic interaction with type I collagen fibrils. J Struct Biol.

[141] Coutinho E., Cardoso M.V., Fernandes C.P., Neves A.A., Gouvea C.V., Van Landuyt K.L. (2011). Nanoleakage distribution at adhesive–dentin interfaces in 3D. J Dent Res.

[142] Ottani V., Martini D., Franchi M., Ruggeri A., Raspanti M. (2002). Hierarchical structures in fibrillar collagens. Micron.

[143] Powers J.M., Sakaguchi R.L. (2006). Craig’s restorative dental materials.

[144] Gautieri A., Vesentini S., Redaelli A., Buehler M.J. (2010). Hierarchical structure and nanomechanics of collagen microfibrils from the atomistic scale up. Nano Lett.

[145] Scott P.G., McEwan P.A., Dodd C.M., Bergmann E.M., Bishop P.N., Bella J. (2004). Crystal structure of the dimeric protein core of decorin, the archetypal small leucine-rich repeat proteoglycan. Proc Natl Acad Sci USA.

[146] Winter W.T., Arnott S., Isaac D.H., Atkins E.D. (1978). Chondroitin 4-sulfate: the structure of a sulfated glycosaminoglycan. J Mol Biol.

[147] Lehmann N., Debret R., Romeas A., Magloire H., Degrange M., Bleicher F. (2009). Self-etching increases matrix metalloproteinase expression in the dentin–pulp complex. J Dent Res.

[148] Mazzoni A., Carrilho M., Papa V., Tjaderhane L., Gobbi P., Nucci C. (2011). MMP-2 assay within the hybrid layer created by a two-step etch-and-rinse adhesive: biochemical and immunohistochemical analysis. J Dent.

